# Supplementary figures and images for: A Y-linked duplication of anti-Mullerian hormone is the sex determination gene in threespine stickleback
Source: PLoS Genet. 2025 Nov 4;21(11):e1011932. doi: 10.1371/journal.pgen.1011932 (PMC12599925; doi:10.1371/journal.pgen.1011932)

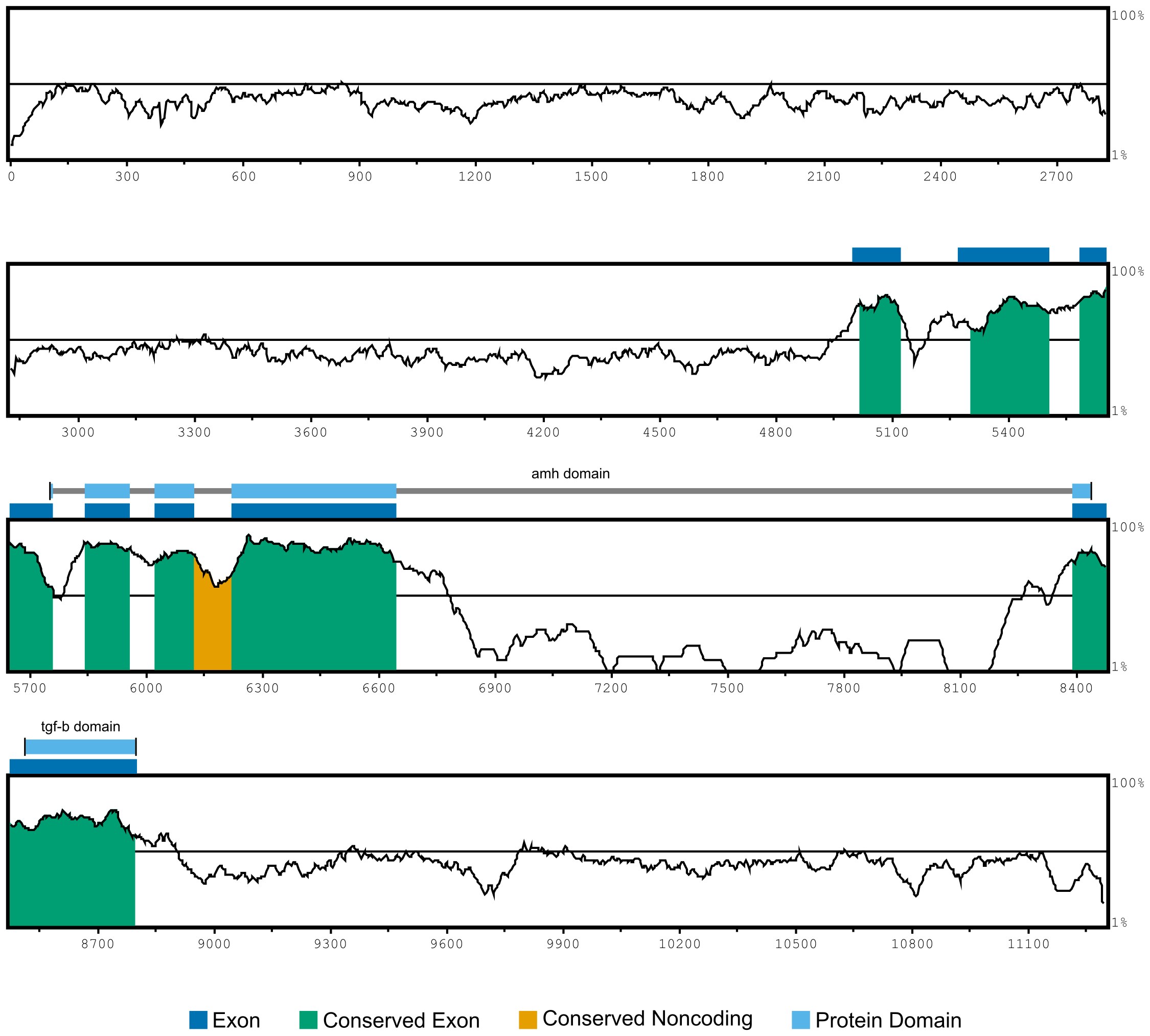

Supplement: S1 Fig — Vistaplot showing conserved sequence identity of amhy to amh with sequence identity on the Y axis and base pair position of amhy on the x axis. Exon positions are indicated in dark blue, and the amh domain and TGF-β domain are indicated in light blue above the graph. Conserved sequences share 70% sequence identity in a 100 bp window. (TIF) [file pgen.1011932.s001.tif]

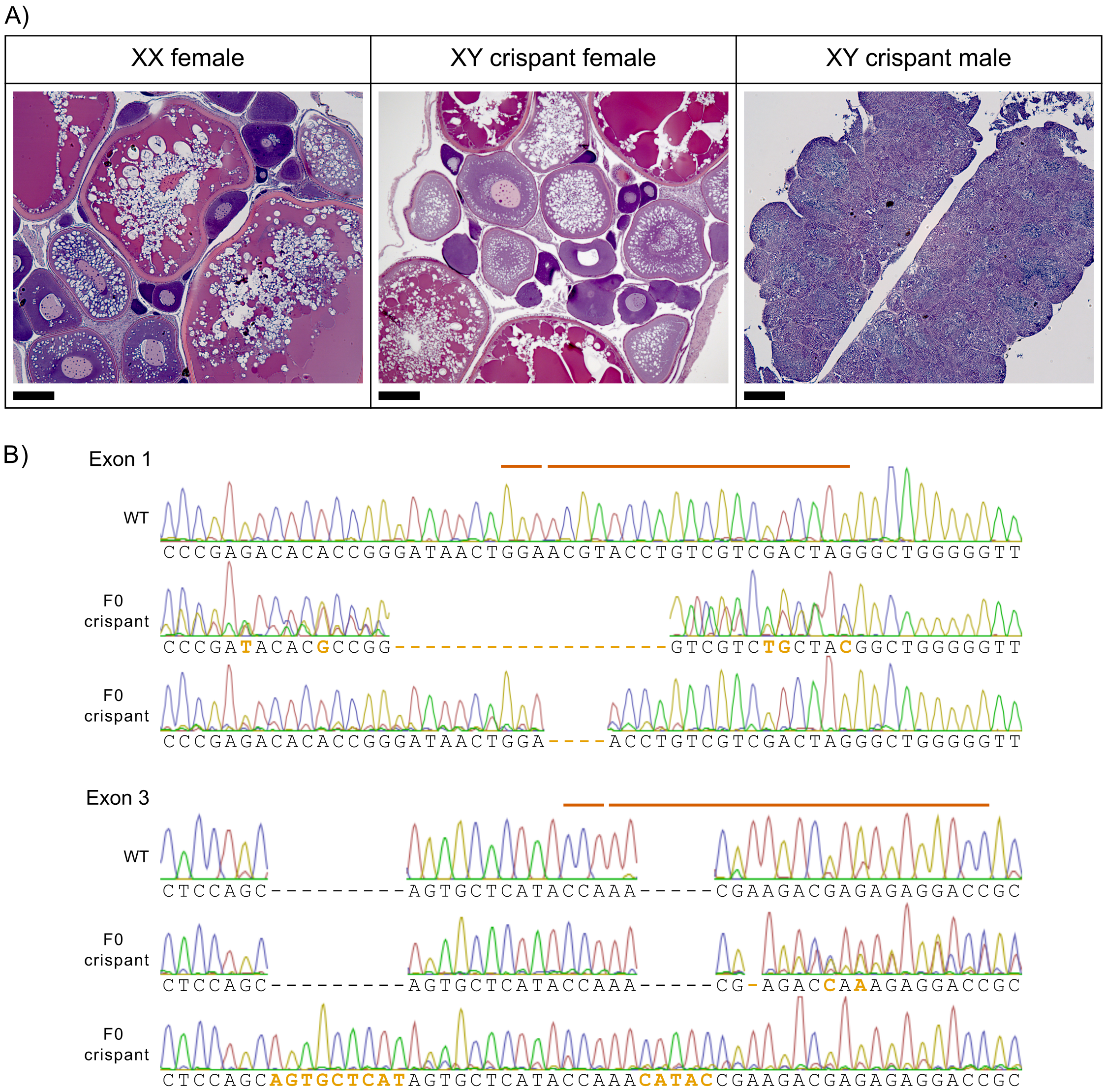

Supplement: S2 Fig — A) XY crispants developed as females with ovaries or males with testes. B) Sanger sequencing of exon 1 and exon 3 show various mutations relative to wildtype. Discordant chromatograms in crispants are indicative of mosaic mutations. Mutations are bolded in orange. CRISPR target site and PAM sequence are indicated by orange lines above wildtype reference sequence. (TIF) [file pgen.1011932.s002.tif]

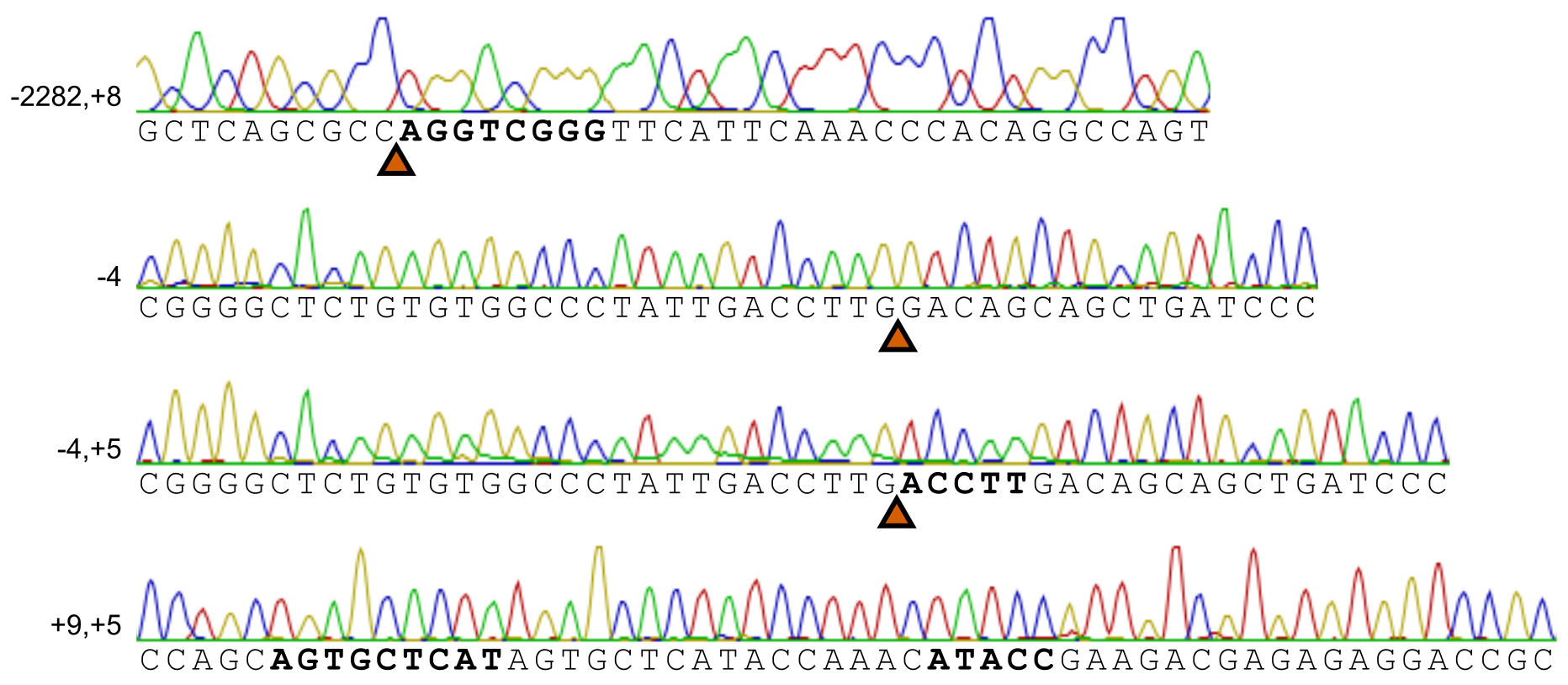

Supplement: S3 Fig — Inserted base pairs are bolded. Locations of deletions are indicated with red arrowheads. (TIF) [file pgen.1011932.s003.tif]

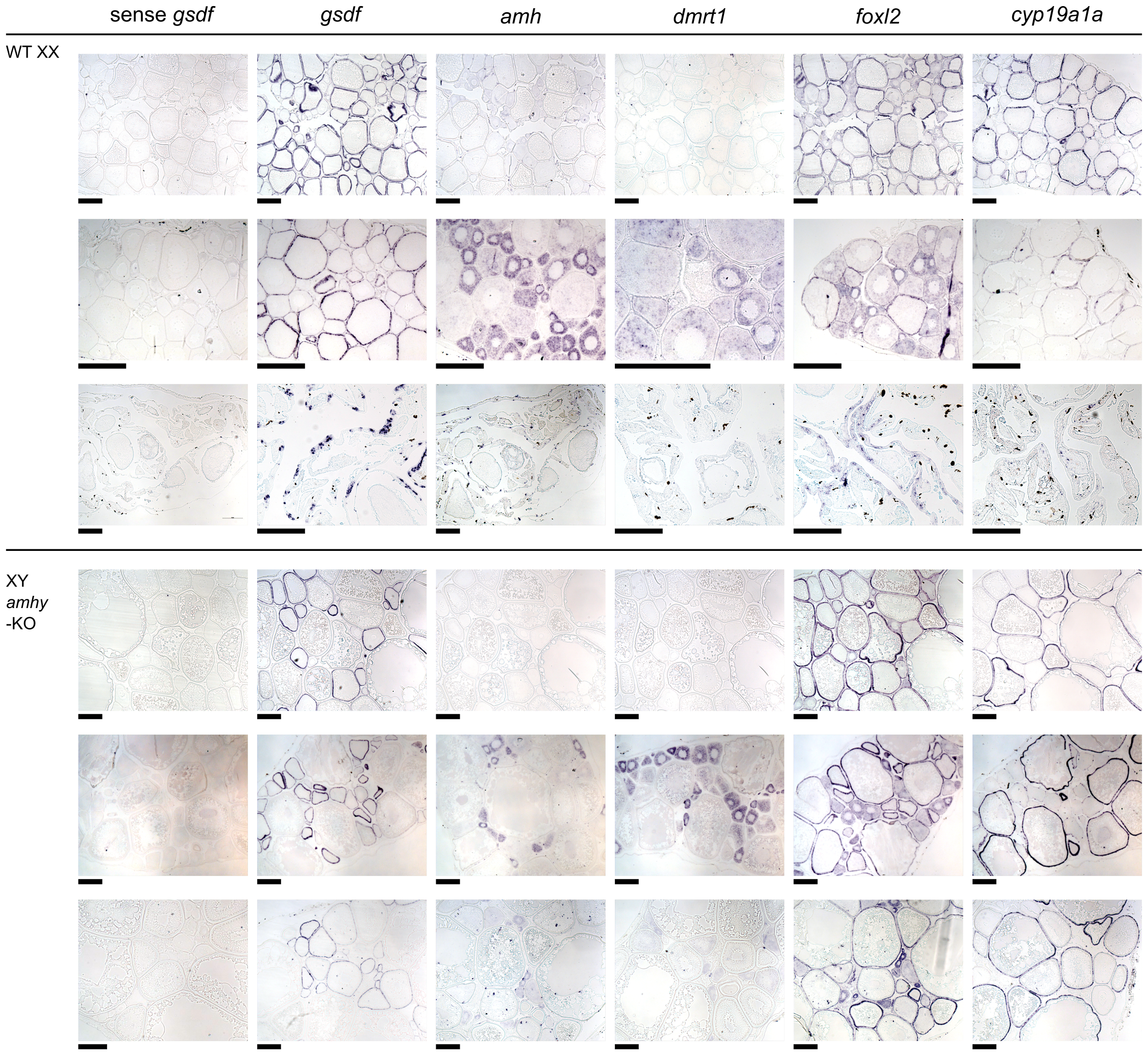

Supplement: S4 Fig — In situ hybridization of gonadal genes in three biological replicates for wildtype XX ovaries and XY amhy-KO ovaries. Scale bars = 200 μm. (TIF) [file pgen.1011932.s004.tif]

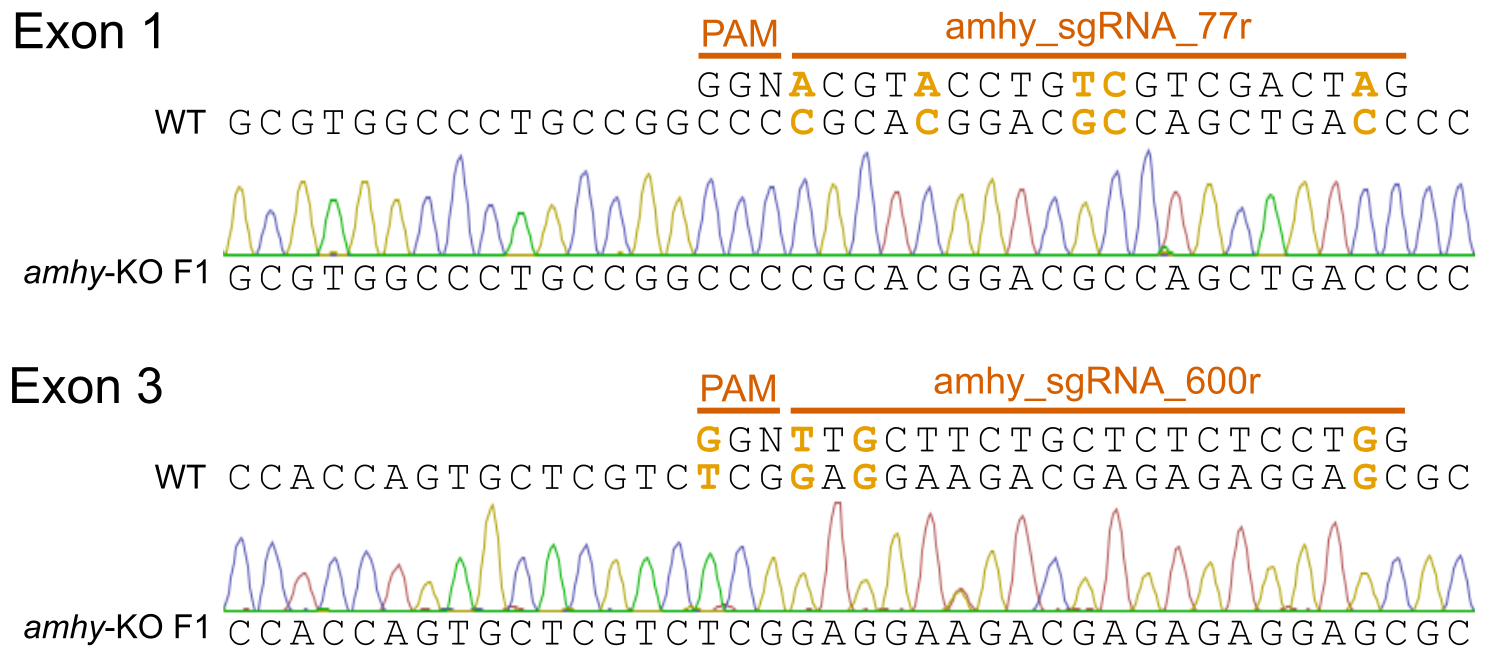

Supplement: S5 Fig — PAM and sgRNA sequences are shown above the reference sequence. Mismatched nucleotides are bolded in orange text. Sanger sequencing of sex-reversed XY F1 amhy-KO fish showed no off-target mutations in amh in exon 1 (A) or exon 3 (B). (TIF) [file pgen.1011932.s005.tif]

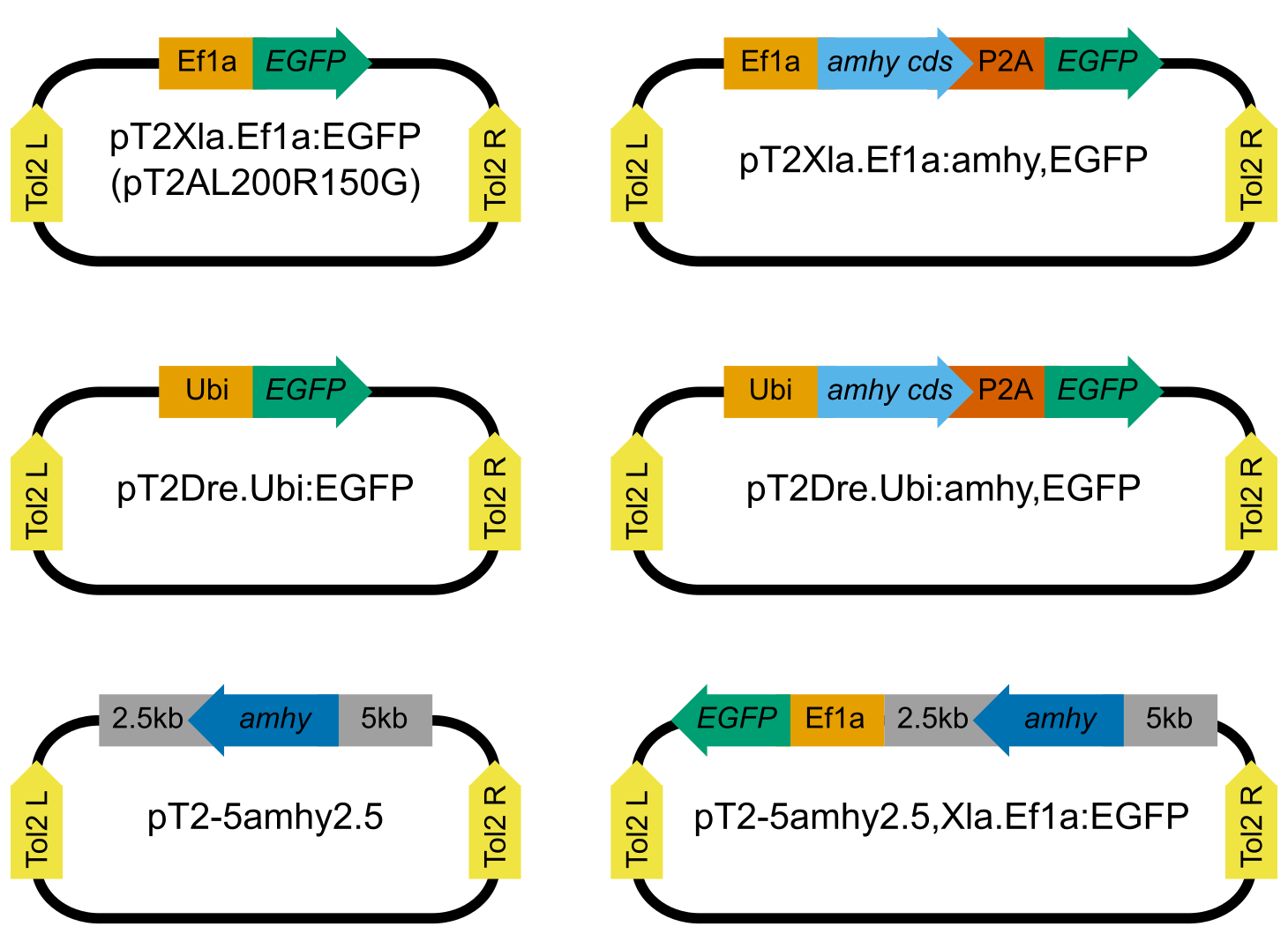

Supplement: S6 Fig — Transgenes used the ectopic X. laevis Ef1a or D. rerio Ubi promoter or the endogenous amhy promoter. Control transgenes express only EGFP. Other transgenes contain the amhy coding sequence or the full amhy sequence including introns. To screen for transgene integration, amhy transgenes include an EGFP joined to amhy with a P2A self-cleaving peptide or separate EGFP cassette. All transgenes are flanked by Tol2 transposon arms to facilitate transgene integration. (TIF) [file pgen.1011932.s006.tif]

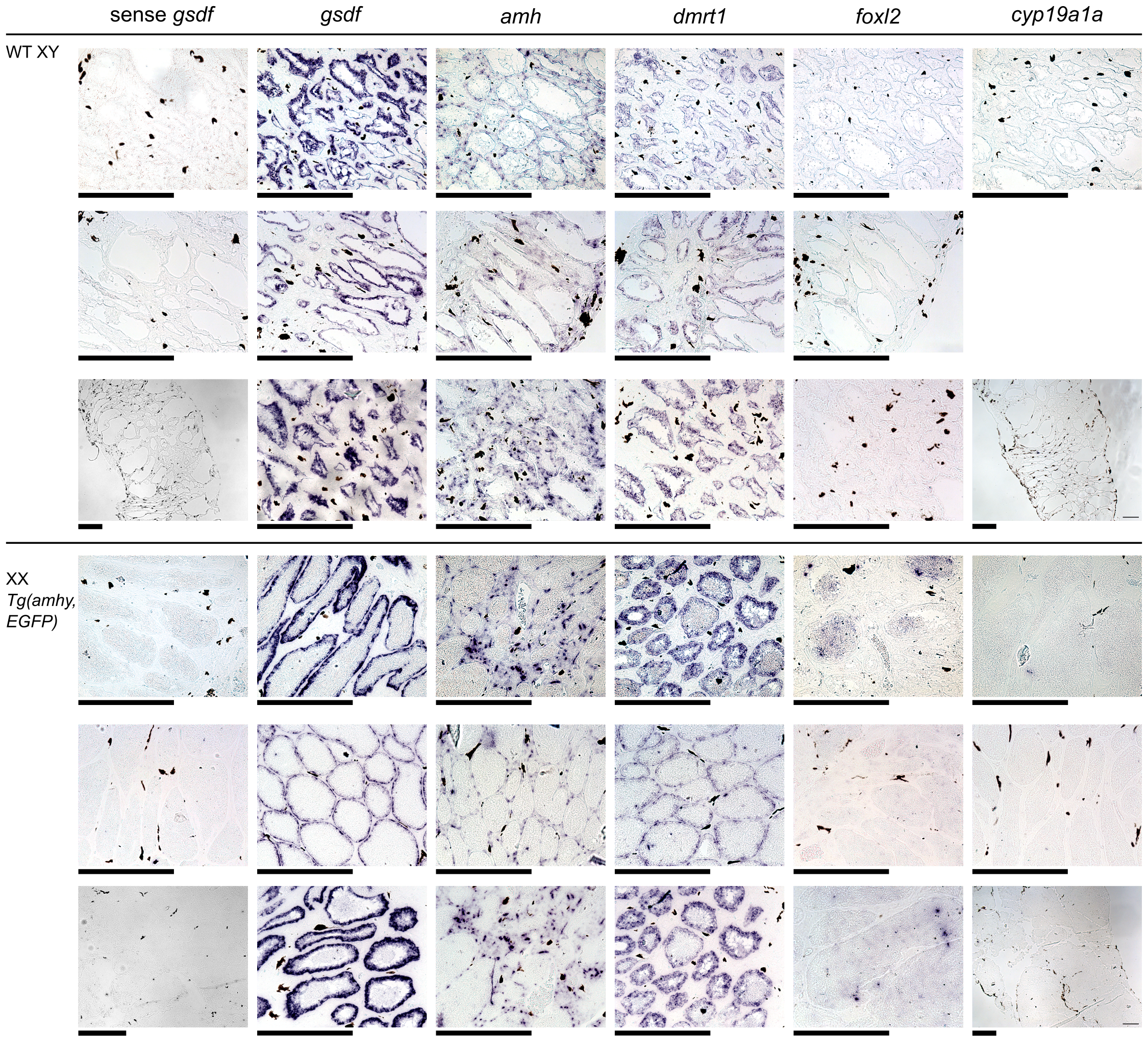

Supplement: S7 Fig — In situ hybridization of gonadal genes in three biological replicates for wildtype XY testes and XX Tg(amhy,EGFP) testes. For one wildtype replicate, the tissue was lost during staining for cyp19a1a. Scale bars = 200 μm. (TIF) [file pgen.1011932.s007.tif]

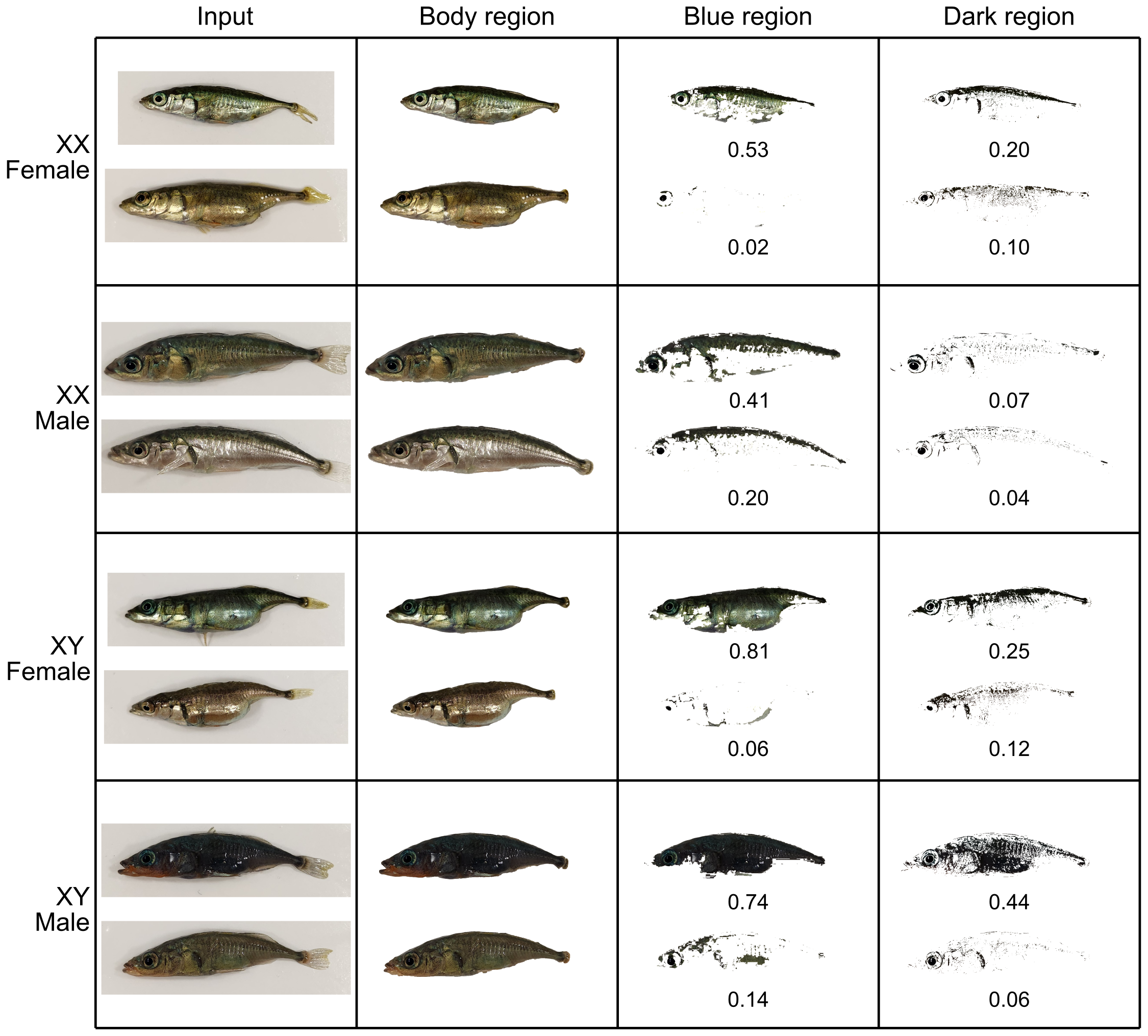

Supplement: S8 Fig — Representative high and low coloration individual for XX wildtype females, XX Tg(amhy,EGFP) males, XY amhy-KO females, and XY wildtype males. Blue and dark regions were quantified for each individual. Coloration proportion values are shown below each individual. (TIF) [file pgen.1011932.s008.tif]

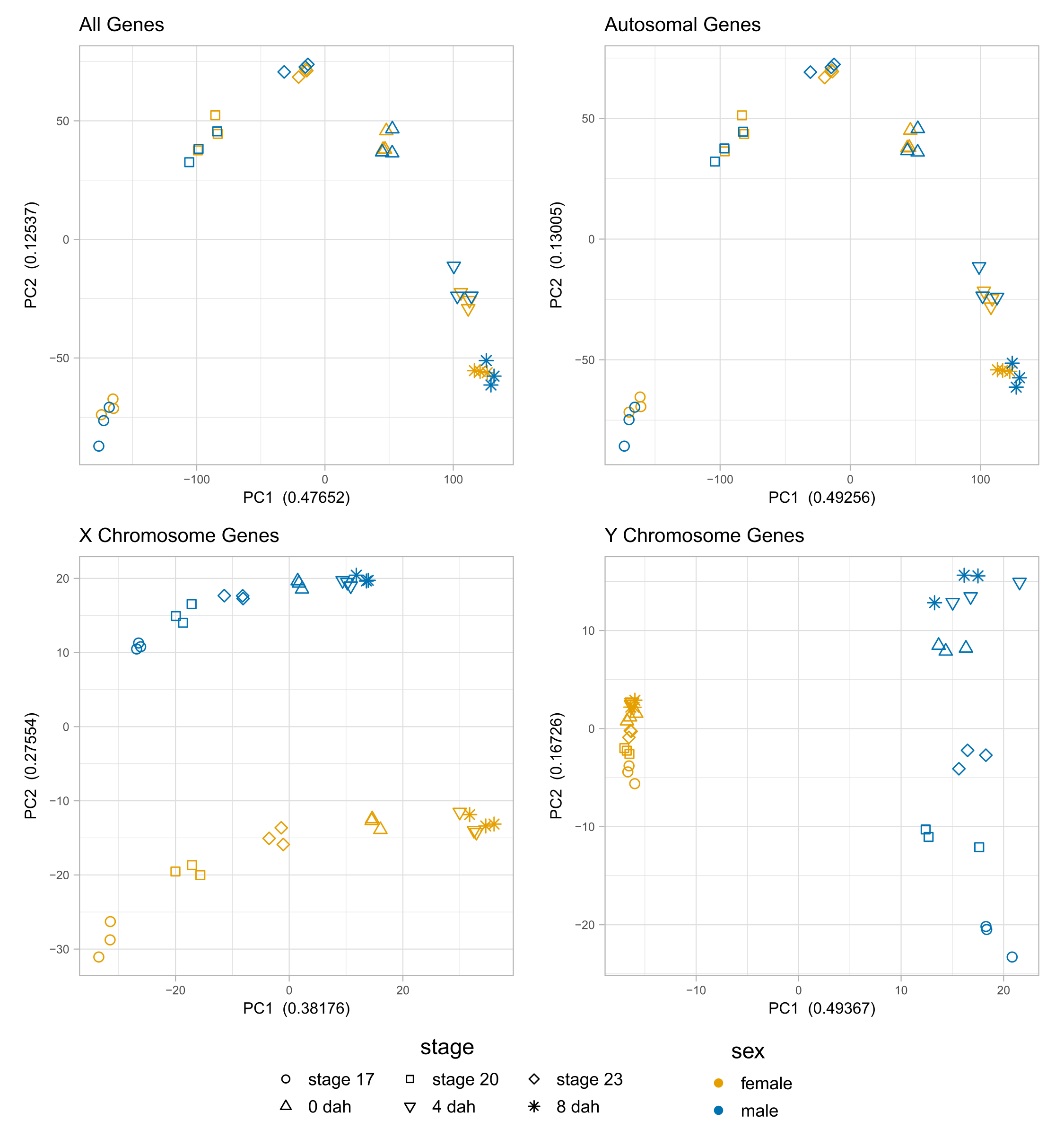

Supplement: S9 Fig — Principal components 1 and 2 with the Proportion of Variance Explained on the X and Y axes, respectively. When analyzing all genes or autosomal genes only, samples group by developmental stage and not sex. X chromosome genes show separation by both stage and sex. Y chromosome genes group primarily by sex with male samples separate by developmental stage. (TIF) [file pgen.1011932.s009.tif]

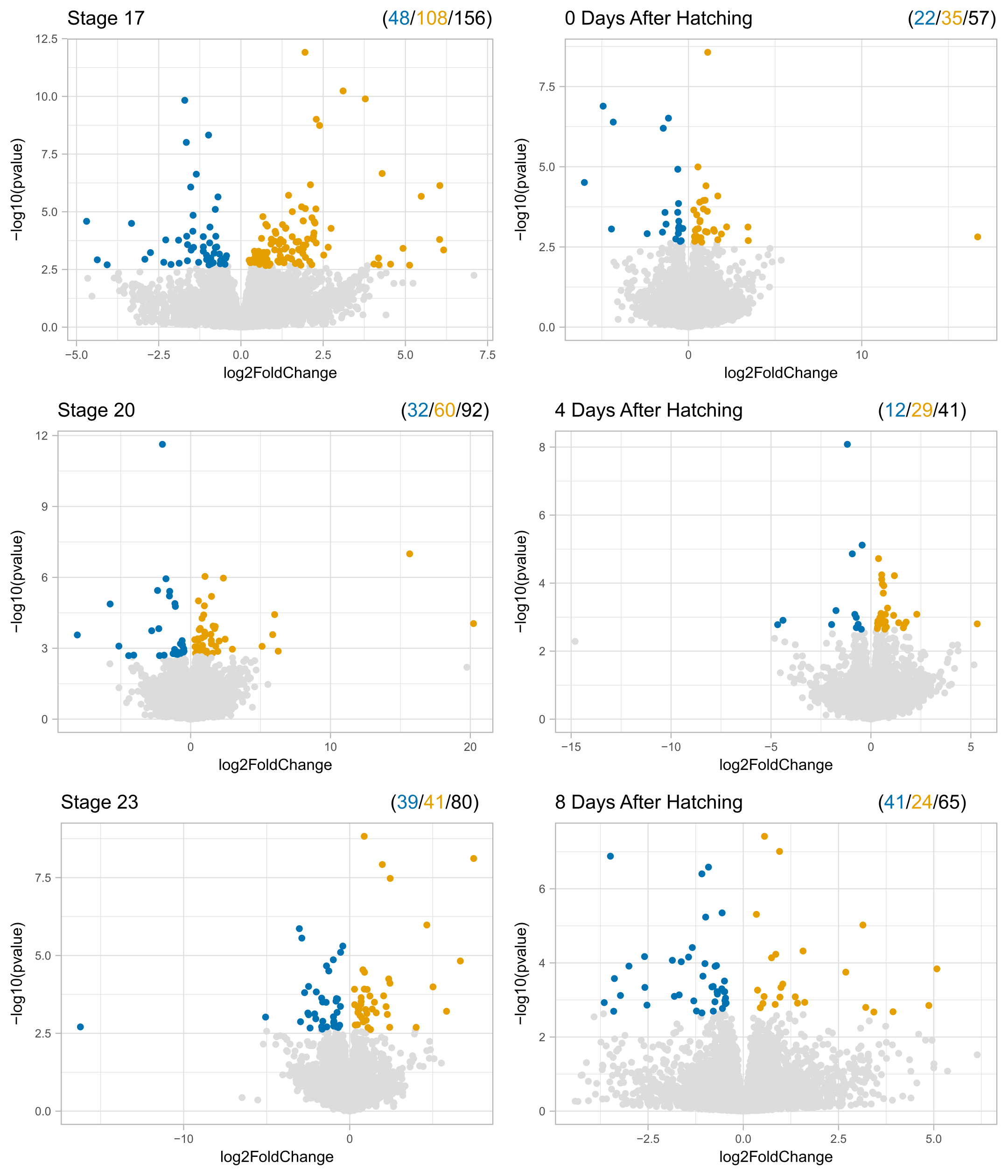

Supplement: S10 Fig — Positive log2 Fold Change values indicate higher expression in females, and negative values indicate higher expression in males. Significant genes (p < 0.05) are shaded. The number of male biased, female biased, and total differentially expressed genes are shown above each plot. (TIF) [file pgen.1011932.s010.tif]
