# Supplementary material for: A Y-linked duplication of anti-Mullerian hormone is the sex determination gene in threespine stickleback
Source: PLoS Genet. 2025 Nov 4;21(11):e1011932. doi: 10.1371/journal.pgen.1011932 (PMC12599925; doi:10.1371/journal.pgen.1011932)
Supplement: S1 Table — (DOCX) [file pgen.1011932.s011.docx]

| Female | Male | Clutch Size | Viable | Hatched | Viable:Total | Hatched:Total | Hatched:Viable |
| --- | --- | --- | --- | --- | --- | --- | --- |
| 1 | A | 95 | 95 | 94 | 1.0000 | 0.9895 | 0.9895 |
| 2 | A | 66 | 65 | 64 | 0.9848 | 0.9697 | 0.9846 |
| 3 | A | 91 | 61 | 60 | 0.6703 | 0.6593 | 0.9836 |
| 4 | B | 110 | 107 | 107 | 0.9727 | 0.9727 | 1.0000 |
| 5 | B | 65 | 58 | 58 | 0.8923 | 0.8923 | 1.0000 |
| 6 | B | 116 | 116 | 116 | 1.0000 | 1.0000 | 1.0000 |
| 7 | C | 107 | 100 | 98 | 0.9346 | 0.9159 | 0.9800 |
| 8 | C | 90 | 54 | 43 | 0.6000 | 0.4778 | 0.7963 |
| 9 | C | 117 | 74 | 68 | 0.6325 | 0.5812 | 0.9189 |
| total | A | 252 | 221 | 218 | 0.8770 | 0.8651 | 0.9864 |
| total | B | 291 | 281 | 281 | 0.9656 | 0.9656 | 1.0000 |
| total | C | 314 | 228 | 209 | 0.7261 | 0.6656 | 0.9167 |
| total | total | 857 | 730 | 708 | 0.8518 | 0.8261 | 0.9699 |
