# Supplementary material for: A Y-linked duplication of anti-Mullerian hormone is the sex determination gene in threespine stickleback
Source: PLoS Genet. 2025 Nov 4;21(11):e1011932. doi: 10.1371/journal.pgen.1011932 (PMC12599925; doi:10.1371/journal.pgen.1011932)
Supplement: S2 Table — (DOCX) [file pgen.1011932.s012.docx]

| Female | Cross Number | Clutch Size | Viable | Hatched | Viable:Total | Hatched:Total | Hatched:Viable |
| --- | --- | --- | --- | --- | --- | --- | --- |
| A | 1 | 43 | 1 | 1 | 0.0233 | 0.0233 | 1.0000 |
| B | 1 | 51 | 2 | 0 | 0.0392 | 0.0000 | 0.0000 |
| C | 1 | 63 | 4 | 2 | 0.0635 | 0.0317 | 0.5000 |
| C | 2 | 99 | 24 | 21 | 0.2424 | 0.2121 | 0.8750 |
| C | 3 | 81 | 11 | 6 | 0.1358 | 0.0741 | 0.5455 |
| C | 4 | 100 | 78 | 54 | 0.7800 | 0.5400 | 0.6923 |
| C | 5 | 89 | 70 | 46 | 0.7865 | 0.5169 | 0.6571 |
