# Supplementary material for: A Y-linked duplication of anti-Mullerian hormone is the sex determination gene in threespine stickleback
Source: PLoS Genet. 2025 Nov 4;21(11):e1011932. doi: 10.1371/journal.pgen.1011932 (PMC12599925; doi:10.1371/journal.pgen.1011932)
Supplement: S3 Table — (DOCX) [file pgen.1011932.s013.docx]

| Genotype | Clutch Size | Viable | Hatched | Viable:Total | Hatched:Total | Hatched:Viable |
| --- | --- | --- | --- | --- | --- | --- |
| XX | 57 | 57 | 57 | 1.0000 | 1.0000 | 1.0000 |
| XX | 112 | 105 | 34 | 0.9375 | 0.3036 | 0.3238 |
| XX | 56 | 55 | 55 | 0.9821 | 0.9821 | 1.0000 |
| XX | 136 | 90 | 47 | 0.6618 | 0.3456 | 0.5222 |
| XX | 94 | 94 | 79 | 1.0000 | 0.8404 | 0.8404 |
| XY | 84 | 6 | 2 | 0.0714 | 0.0238 | 0.3333 |
| XY | 65 | 0 | 0 | 0.0000 | 0.0000 | NA |
| XY | 98 | 0 | 0 | 0.0000 | 0.0000 | NA |
| XY | 22 | 0 | 0 | 0.0000 | 0.0000 | NA |
| XY | 72 | 6 | 4 | 0.0833 | 0.0556 | 0.6667 |
| XY | 92 | 0 | 0 | 0.0000 | 0.0000 | NA |
| Total XX | 455 | 401 | 272 | 0.8813 | 0.5978 | 0.6783 |
| Total XY | 433 | 12 | 6 | 0.0277 | 0.0139 | 0.5000 |
