# Supplementary material for: A Y-linked duplication of anti-Mullerian hormone is the sex determination gene in threespine stickleback
Source: PLoS Genet. 2025 Nov 4;21(11):e1011932. doi: 10.1371/journal.pgen.1011932 (PMC12599925; doi:10.1371/journal.pgen.1011932)
Supplement: S4 Table — (DOCX) [file pgen.1011932.s014.docx]

| Comparison | Estimate | Standard Error | z.ratio | p.value |
| --- | --- | --- | --- | --- |
| Blue proportion XXWT-XXTG | -0.0951 | 0.0593 | -1.606 | 0.3754 |
| Blue proportion XXWT-XYKO | -0.2144 | 0.0793 | -2.702 | **0.0348** |
| Blue proportion XXWT-XYWT | -0.3622 | 0.0738 | -4.906 | **<0.0001** |
| Blue proportion XXTG-XYKO | -0.1192 | 0.0718 | -1.66 | 0.3451 |
| Blue proportion XXTG-XYWT | -0.2671 | 0.0657 | -4.066 | **0.0003** |
| Blue proportion XYKO-XYWT | -0.1478 | 0.0843 | -1.754 | 0.2956 |
| Dark proportion XXWT-XXTG | 0.0458 | 0.0197 | 2.322 | 0.093 |
| Dark proportion XXWT-XYKO | -0.0563 | 0.0249 | -2.26 | 0.1074 |
| Dark proportion XXWT-XYWT | -0.1047 | 0.047 | -2.226 | 0.1161 |
| Dark proportion XXTG-XYKO | -0.1021 | 0.0266 | -3.834 | **0.0007** |
| Dark proportion XXTG-XYWT | -0.1505 | 0.0479 | -3.138 | **0.0092** |
| Dark proportion XYKO-XYWT | -0.0484 | 0.0503 | -0.961 | 0.7716 |
