# Supplementary material for: A Y-linked duplication of anti-Mullerian hormone is the sex determination gene in threespine stickleback
Source: PLoS Genet. 2025 Nov 4;21(11):e1011932. doi: 10.1371/journal.pgen.1011932 (PMC12599925; doi:10.1371/journal.pgen.1011932)
Supplement: S5 Table — (DOCX) [file pgen.1011932.s015.docx]

| Threespine *amh* and threespine *amhy* | | | | | | | | | | | |
| --- | --- | --- | --- | --- | --- | --- | --- | --- | --- | --- | --- |
|  | Exon 1 | Exon 2 | Exon 3 | Exon 4 | Exon 5 | Exon 6 | Exon 7 | CDS | Introns | 2kb Upstream | 2kb Downstream |
| Sequence  identity | 0.760 | 0.682 | 0.812 | 0.866 | 0.790 | 0.846 | 0.742 | 0.780 | 0.286 | 0.365 | 0.376 |
| dN/dS | 0.583 | 0.362 | 0.512 | 0.803 | 0.651 | 0.373 | 0.723 | 0.534 | - | - | - |
| Threespine *amh* and ninespine *amh* | | | | | | | | | | | |
|  | Exon 1 | Exon 2 | Exon 3 | Exon 4 | Exon 5 | Exon 6 | Exon 7 | CDS | Introns | 2kb Upstream | 2kb Downstream |
| Sequence  identity | 0.791 | 0.769 | 0.890 | 0.920 | 0.870 | 0.905 | 0.936 | 0.879 | 0.668 | 0.595 | 0.660 |
| dN/dS | 0.597 | 0.425 | 1.003 | 0.441 | 0.266 | 0.565 | 0.278 | 0.467 | - | - | - |
