# Supplementary material for: A Y-linked duplication of anti-Mullerian hormone is the sex determination gene in threespine stickleback
Source: PLoS Genet. 2025 Nov 4;21(11):e1011932. doi: 10.1371/journal.pgen.1011932 (PMC12599925; doi:10.1371/journal.pgen.1011932)
Supplement: S6 Table — Length adjusted amhy counts were calculated by multiplying amhy normalized read counts by transcript length of amh (3895 bp) divided by amhy (2496 bp). P values show two tailed Welch’s t-test for a difference between amhy and one half amh expression in males at each stage and one tailed Welch’s t-test for a greater total amh dosage in males than in females at each stage. Significant values are bolded (p < 0.05). (DOCX) [file pgen.1011932.s016.docx]

| stage | stage 17 | | stage 20 | | stage 23 | | 0 dph | | 4 dph | | 8 dph | |
| --- | --- | --- | --- | --- | --- | --- | --- | --- | --- | --- | --- | --- |
| sex | female | male | female | male | female | male | female | male | female | male | female | male |
| *amh* normalized counts | 24.08 | 28.94 | 15.13 | 34.03 | 32.04 | 17.02 | 18.72 | 37.12 | 6.94 | 12.47 | 17.04 | 23.57 |
|  | 34.14 | 13.17 | 22.49 | 18.61 | 17.94 | 12.90 | 29.62 | 14.87 | 7.01 | 10.98 | 12.34 | 14.82 |
|  | 27.42 | 25.48 | 32.20 | 17.49 | 16.36 | 24.01 | 18.31 | 23.89 | 17.46 | 6.78 | 10.36 | 19.66 |
| Mean | 28.55 | 22.53 | 23.27 | 23.38 | 22.11 | 17.98 | 22.22 | 25.29 | 10.47 | 10.08 | 13.25 | 19.35 |
| SD | 5.12 | 8.29 | 8.56 | 9.24 | 8.64 | 5.62 | 6.41 | 11.19 | 6.06 | 2.95 | 3.43 | 4.38 |
| *amhy* normalized counts | 0.00 | 8.40 | 0.00 | 0.95 | 0.00 | 13.62 | 0.85 | 19.88 | 0.00 | 37.42 | 0.00 | 13.60 |
|  | 0.00 | 10.13 | 0.00 | 4.65 | 0.00 | 8.60 | 0.00 | 20.12 | 0.00 | 20.58 | 0.00 | 33.13 |
|  | 0.00 | 2.83 | 0.00 | 2.06 | 0.00 | 11.08 | 2.93 | 8.76 | 0.00 | 16.27 | 0.00 | 9.44 |
| Mean | 0.00 | 7.12 | 0.00 | 2.55 | 0.00 | 11.10 | 1.26 | 16.25 | 0.00 | 24.76 | 0.00 | 18.72 |
| sd | 0.00 | 3.81 | 0.00 | 1.90 | 0.00 | 2.51 | 1.51 | 6.49 | 0.00 | 11.18 | 0.00 | 12.65 |
| length adjusted *amhy* counts | 0.00 | 13.11 | 0.00 | 1.48 | 0.00 | 21.25 | 1.33 | 31.03 | 0.00 | 58.40 | 0.00 | 21.22 |
|  | 0.00 | 15.81 | 0.00 | 7.26 | 0.00 | 13.42 | 0.00 | 31.40 | 0.00 | 32.11 | 0.00 | 51.70 |
|  | 0.00 | 4.42 | 0.00 | 3.21 | 0.00 | 17.29 | 4.57 | 13.67 | 0.00 | 25.39 | 0.00 | 14.73 |
| Mean | 0.00 | 11.11 | 0.00 | 3.98 | 0.00 | 17.32 | 1.97 | 25.37 | 0.00 | 38.63 | 0.00 | 29.22 |
| SD | 0.00 | 5.95 | 0.00 | 2.97 | 0.00 | 3.91 | 2.35 | 10.13 | 0.00 | 17.44 | 0.00 | 19.74 |
| P *amhy* ≠ 0.5**amh* | - | 0.9730 | - | 0.0830 | - | **0.0454** | - | 0.1496 | - | 0.0783 | - | 0.2275 |
| *amh +* length adj. *amhy* counts | 24.08 | 42.05 | 15.13 | 35.51 | 32.04 | 38.27 | 20.05 | 68.15 | 6.94 | 70.87 | 17.04 | 44.78 |
|  | 34.14 | 28.98 | 22.49 | 25.86 | 17.94 | 26.32 | 29.62 | 46.27 | 7.01 | 43.09 | 12.34 | 66.53 |
|  | 27.42 | 29.89 | 32.20 | 20.71 | 16.36 | 41.31 | 22.88 | 37.56 | 17.46 | 32.16 | 10.36 | 34.39 |
| Mean | 28.55 | 33.64 | 23.27 | 27.36 | 22.11 | 35.30 | 24.19 | 50.66 | 10.47 | 48.71 | 13.25 | 48.57 |
| SD | 5.12 | 7.30 | 8.56 | 7.51 | 8.64 | 7.92 | 4.91 | 15.76 | 6.06 | 19.96 | 3.43 | 16.40 |
| P total *amh* males > females | 0.1922 | | 0.2843 | | 0.0618 | | **0.0448** | | **0.0347** | | **0.0297** | |
