# Supplementary material for: A Y-linked duplication of anti-Mullerian hormone is the sex determination gene in threespine stickleback
Source: PLoS Genet. 2025 Nov 4;21(11):e1011932. doi: 10.1371/journal.pgen.1011932 (PMC12599925; doi:10.1371/journal.pgen.1011932)
Supplement: S7 Table — (DOCX) [file pgen.1011932.s017.docx]

| Assay | Forward primer | Reverse primer |
| --- | --- | --- |
| XY *idh* genotyping | 5’-GGGACGAGCAAGATTTATT-3' | 5’-TTATCGTTAGCCAGGAGATGG-3' |
| *amhy* exon 1 genotyping, *amhy* transgene detection | 5’-TTGGTGGGTTGATGCTCTGC-3' | 5’-CTATGTGAGAGACTGTGGCGG-3' |
| *amhy* exon 3 genotyping | 5’-TCAGTTTGGAATCTGCACTGTT-3' | 5’-GGATGCAGTGATTTACTGGTGA-3' |
| *amhy* 2.3 kb deletion genotyping | 5’-CACAGTCCTTCGACTCCTTCG-3' | 5’-GAGACCATGGTGACTGGCTC-3' |
| *amh* exon 1 genotyping | 5’-GGCAGTGAGAAGGGAAGGTG-3' | 5’-GGCCTCTGGTGATGGGAATG-3' |
| *amh* exon 3 genotyping | 5’-CAGCCGGAGTCAGTTTGGAA-3' | 5’-TCATGCATCGTCGACTGGAG-3' |
| EGFP transgene detection | 5’-ATCATGGCCGACAAGCAGAA-3' | 5’-AACTCCAGCAGGACCATGTG-3' |
| *dmrt1* riboprobe cloning | 5’-AGCAGCACCGAGAAGCAG-3’ | 5’-CTCGTTGACGTTGCCCATGA-3’ |
| *gsdf* riboprobe cloning | 5’-TTTTGTCACGGCGATGCTTC-3’ | 5’-AGGAAATGACGAGGGTGCTG-3’ |
| *foxl2* riboprobe cloning | 5’-GAGAAGGTCCCGGAGAAAGC-3’ | 5’-GCAGTGCATCATCGAGAGCT-3’ |
| *cyp19a1a* riboprobe cloning | 5’-CCTTGTTTCTGTCTGGGCCT-3’ | 5’-TTGAGGAGCAGCAGCATGA -3’ |
| Riboprobe template synthesis | 5’-TAATACGACTCACTATAGGG-3’ | 5’-TAATACGACTCACTATAGGG-3’ |
