# Supplementary material for: A Y-linked duplication of anti-Mullerian hormone is the sex determination gene in threespine stickleback
Source: PLoS Genet. 2025 Nov 4;21(11):e1011932. doi: 10.1371/journal.pgen.1011932 (PMC12599925; doi:10.1371/journal.pgen.1011932)
Supplement: Attachment — Submitted filename: Response for reviewers.docx [file pgen.1011932.s021.docx]

Manuscript PGENETICS-D-25-00679

Response to reviewers

Dear Editor,

Thank you and the reviewers for your time spent reviewing our paper and providing your feedback. The authors have carefully considered all comments and have tried our best to address them. We welcome any further recommendations on this manuscript.

We wish to highlight one major change to the transcriptomic data in this study: Upon manual inspection of transcriptomic data of *amh* and *amhy*, we identified an unannotated transcript downstream of *amh* in the opposite orientation, such that the 3’ end of this transcript overlaps slightly with *amh*. Reads from this gene were being incorrectly assigned to *amh* and artificially inflating expression values. By modifying how read strandedness was handled during the counting step, we were able to remove these erroneous reads (see methods, lines 846-852).

This alteration led to a dramatic reduction of *amh* counts at later stages of development and the novel finding that *amhy* contributes to a higher overall amh dosage at later stages of development (see lines 374-378, S6 Table). This change in methodology resulted in small changes in the number of DEGs at each stage and logFC and p-values for differential expression which have all been updated. There were no changes in the statistical significance for differential expression or noteworthy changes in read count for any named genes of interest other than *amh*.

Please see the point-by-point response to the reviewer’s comments below. All line numbers refer to the revised manuscript file with tracked changes.

Reviewer #1: Treaster and colleagues investigated the genetic basis for sex determination in threespine stickleback (Gac) fish. Amh has evolved independently as a sex determining gene in multiple vertebrate species. The amh gene has been duplicated onto the Y chromosome in the threespine stickleback. Amhy was mutated in XY Gac fish by CRISPR/Cas, resulting in completely sex-reversed male-to-females. Conversely, a genomic fragment of amhy introduced into XX Gac fish by transposons led to female-to-male sex reversed fish. Thus, amhy is both necessary and sufficient for sex determination in Gac fish. These mutants also provided the opportunity to explore fertility, YY embryos, and hormonal/sex chromosome dependent phenotypes.

Pg 16, 399-402 regarding ancestral role of amh for germ cells. Transgenic mice overexpressing human AMH have primordial follicles at birth, but these are subsequently lost and the remaining somatic tissues form seminiferous tubulelike structures (Behringer 1990). Perhaps this can be included in the discussion.

In mice, Durlinger (1999), showed that Amh regulated the recruitment of primordial follicles.

Author Response: Thank you for bringing these sources to our attention. We have incorporated them into our discussion on the function of amh.

Line 481: Even after primary sex determination, germ cell depletion in mice [77] and rats [108] leads to masculinization of the ovary, which produces Sertoli-like cells and seminiferous tubule-like structures before its eventual degradation.

Line 495: Overexpression of *Amh* in mice [77,109] causes reduced germ cell count and gonad size in males and females, and loss of Amh alters primordial follicle recruitment in females [110].

Pg 8, 194-204. The authors attempted to generate amh overexpressing fish but were not successful at the level of GFP expression, leading them to speculate that extremely high and widespread expression of amh is cytotoxic. However, in mice overexpression of human AMH is compatible with life (Behringer 1990).

Author Response: Thank you for providing this information. After additional reading, we agree that cytotoxicity of amh seems unlikely. We have revised our interpretation of these results as follows:

Line 226: *Amh* has been overexpressed successfully in mouse [77], chicken [78], and Nile tilapia [16], and the P2A peptide has been successfully used in stickleback overexpression constructs [79], indicating that overexpression alone or the presence of a P2A peptide was likely not responsible for the failure of this construct. One possibility is introns of *amhy* may contain essential functional elements needed for gene expression of *amhy* in threespine stickleback. The presence of introns has been shown to impact gene expression, even under the regulation of non-native constitutive promoters [80,81].

I am curious what is the knockout phenotype of amha Gac fish. I am not asking for the experiment, but it would be nice to have this discussed.

Author Response: We agree that this is an interesting experiment which we hope to address in the future. We have discussed this experiment and possible outcomes in the discussion:

Line 542: Manipulating *amh* and *amhrII* could provide further insight into the evolution of *amhy* and its mechanism of inducing male development. We would predict knockout of *amh* or *amhrII* to result in over proliferation of germ cells and some level of male to female sex reversal in XY fish as is seen in medaka *amhrII* mutants [32]. This would demonstrate that germ cell proliferation regulated by the canonical *amh* signaling pathway can influence sex in stickleback, making protein evolution or interaction with non-canonical receptors less parsimonious avenues by which *amhy* has evolved its novel function. Misexpression of *amh* and *amhy* using regulatory elements from the opposite gene (i.e. expressing the *amh* genic sequence with *amhy* regulatory elements) or non-native promoters could also elucidate whether coding sequence evolution is important to the function of *amhy*.

Some of the panels in some of the figures would benefit from a white balance of the background.

Author Response: We have improved the white balance on Figures 2, 3, 4, S4, S7.

Reviewer #2: The threespine stickleback is a key model organism for studying evolutionary mechanisms. However, the genetic basis of sex determination in this species has remained unknown. In this study, the authors identify a Y-linked
duplicate of the gene encoding anti-Müllerian hormone (amh), which they name amhy. Duplications of amh or its receptor, amhr, have independently evolved as master sex-determining genes in several vertebrate lineages, making amhy a strong candidate for the sex-determining gene in stickleback. To test this hypothesis, the authors used CRISPR/Cas9-mediated gene knockout and transgenic overexpression approaches. They found that amhy is necessary for male development, as all XY individuals lacking amhy developed as females. Conversely, many XX individuals with ectopic amhy expression showed apparently complete female-to-male sex reversal, indicating that amhy is also sufficient to initiate male development and production of functional testes. These findings strongly support the role of amhy as the primary sex-determining gene in threespine stickleback. The authors then examined the expression of amha and amhy during the developmental window when sex determination is thought to occur. Surprisingly, they found no significant upregulation of amhy relative to its autosomal paralog, nor a marked difference in total amh expression between males and females during this period. This suggests that sex determination may depend on subtle, cell-specific expression patterns or evolutionary changes in the protein-coding sequence. Finally, by analyzing sex-reversed lines, the authors argue that a classic sexually dimorphic trait —blue male nuptial coloration—is influenced both by Y-linked genetic factors and by hormones, independent of sex chromosome genotype. Overall, the data support the hypothesis that amhy is the primary sex determinant in the threespine stickleback. However, there are a few issues that need to be addressed before this analysis is complete.

Major comments:

There are two major issues that need to be addressed. First, there is a lack of controls in several of the data sets presented. These are detailed in the line-by-line critique below. Second, the timepoints chosen for the RNA-seq analysis were expected to cover the window when sex determination had initiated. However, the data shows little to no sex-specific expression of genes that are known sex-specific genes in a wide number of vertebrate species. This could be due to 1) the assumed timing of sex determination is inaccurate (see point below about rearing conditions), and/or 2) the method of analysis was not sensitive enough to detect differences. To this point, estimating gene expression levels from data generated from whole animal RNA will not be as accurate as using RNA isolated from only gonads. If it is not possible to isolate gonads from these early stages (it should be after some practice), then perhaps it would be more informative to analyze gene expression for the canonical sex-specific genes use RNA in situ on sections isolated gonads or on sections where gonads can be identified. In short, the conclusions drawn from the current gene expression analysis are the weakest part of this otherwise compelling manuscript.

Author Response: We agree that our RNA-seq analysis would be more accurate if we could isolate only gonad tissue at this early stage of development. Unfortunately, we have not been able to do so at the time points in this study, as the gonads are extremely small and friable. These difficulties match development in other teleost fish. For comparison, zebrafish (<https://doi.org/10.3791/55294>) and medaka (<https://zoores.ac.cn/en/article/doi/10.11813/j.issn.0254-5853.2013.5.0464>) gonads can only be dissected from comparatively later developmental stages.

However, we believe the lack of substantial sex differentiation in gene expression at these early developmental stages is not driven completely by the lack of sensitivity from using whole embryos. For example, we detected significant female biased expression of *hsd17b1* and *amhrII* which are among the earliest genes differentially expressed in the gonads of sturgeon and mouse, respectively. We also detected significant female biased expression of gonad-specific germ cell genes, *ddx4* and *piwil1*. Differential expression of these genes aligns with the timing of early proliferation of germ cells in females outlined by Lewis et al. 2008. Stage 26, time of hatching, was also similar between our study (200 hpf) and Lewis et al. 2008 (8 dpf), suggesting that our rearing conditions did not change the timing of sex determination and differentiation (see later response on rearing conditions).

Based on these results, we believe the best explanation is that much of sex differentiation occurs at stages later than we collected. We note that later developmental stages need to be analyzed to test this:

Line 567: Female biased expression of *hsd17b1* precedes differential expression of these canonical differentiation genes in sturgeon, where *hsd17b1* is thought to increase estrogen, upregulating of *foxl2* and *cyp19a1a* in females [91]. In stickleback, female biased *hsd17b1* expression may be a key difference between males and females, initiating expression changes in canonical differentiation genes. Further analysis of gene expression at later timepoints will be necessary to determine if this is the case.

Minor comment/suggestion:

It is not necessary to refer to autosomal amh as amha and could be interpreted to imply that there is an amhb. In other fish, “a” and “b” are reserved for ohnologs that resulted from the teleost-specific whole genome duplication. By contrast, amh and amhy are most likely paralogs that resulted from a small-scale duplication/translocation. Also, precedent in medaka to refer to autosomal dmrt1 as dmrt1 and Y-linked as dmy.

Author Response: We have removed the *amha* designation from the text to avoid confusion.

Lin-by-line comments/suggestions:

Line 64 This sentence is awkward.

Author Response: This sentence has been removed as a part of edits to highlight Y chromosome function and evolution based on suggestions from reviewer 3.

Line 149: “…these distal portions OF amhy,…”; “…but PCR PRIMERS spanning…”

Author Response: Corrected

Line 172: Does “PG female” refer to Port Gardner Bay population fish? If so, then for clarity please define this is Line 132 when Port Gardner is first mentioned.

Author Response: Thank you for this question. A previous draft discussed Pacific Ocean and Port Gardner populations separately, but this was changed as there were no phenotypic differences between populations. We have removed this instance where population designation was mistakenly left in at line 195.

Lines 194-205: This is a negative result that does not add to the main results of the paper. I suggest cutting this section.

Author Response: While we acknowledge that these negative results do not contribute to our central hypothesis, we still feel our failed attempts at creating constructs with these ubiquitous reporters are worth reporting for the stickleback community, where a growing number of research groups are using functional genetics approaches. To highlight the usefulness, we have now included some additional insights from these results:

Line 221: Somatic EGFP expression was observed with both control transgenes indicating these commonly used vertebrate constitutive promoters can drive expression in stickleback.

Line 229: One possibility is introns of *amhy* may contain essential functional elements needed for gene expression of *amhy* in threespine stickleback. The presence of introns has been shown to impact gene expression, even under the regulation of non-native constitutive promoters [80,81].

Line 211: “…hours after fertilization (haf).” Is it not more common to use “hours post-fertilization (hpf)?” OK if haf is standard nomenclature for sticklebacks.

Author Response: Thank you for this suggestion. We have replaced haf with hpf throughout the document.

Line 213: How do these fertility/survival rates compare to wild-type control crosses? Please add control cross numbers to Table S1.

Author Response: We have added data for wildtype control crosses to this section and in table S2.

Line 240: One male was extremely subfertile and only 3% of embryos were viable (n=911). The remaining three males showed normal viability rates (80% n=97; 94% n=89, 98% n=235), similar to viability rates of three wildtype males (73% n=314, 87% n=252, 97% n=291) (S1 Table).

Line 215: This sentence is awkward. Perhaps simply state that ¾ of the arrested fish are genotypically YY.

Author Response: We have clarified this sentence:

Line 266: 36 of the 48 arrested embryos were YY and carried both the paternally transmitted Y chromosome (Y^+^) and the maternally transmitted *amhy*-KO Y chromosome (Y^KO^) (Fig 4D).

Line 224: “Subfertility also extended to second generation…” Based on the hypothesis, it would be expected that all sex reversed females would be sub fertile, regardless of generation. The point of this statement is therefore not clear.

Author Response: We agree that this is an expected outcome. We have changed this sentence to emphasize the benefit of analyzing an F2 cross: comparing sex reversed XY females to full sibling wildtype XX females.

Line 276: We also measured fertility of an F2 cross which contained sex reversed XY females and wildtype XX female siblings.

Line 228: “Two XY F2 females were also eggbound…” There are many reasons fish get eggbound. Without a direct comparison to the frequency with which wild-type fish become eggbound (i.e. comparison to a control group), it is not clear what point the authors are trying to make.

Author Response: We have updated this section to highlight the comparison between the F2 XX and XY females and explicitly stated that no XX females were eggbound.

Line 280: Two F2 females were also eggbound and were unable to be stripped of eggs. Both eggbound females were XY^KO^, and no eggbound XX females were observed at the time crosses were performed.

Line 232 “…and four.” Typo. Likely needs to be deleted.

Author Response: Corrected.

Line 234: Please add wild-type control numbers for comparison and also state numbers as “% viability (n= )” as this is the most relevant number to compare.

Author Response: We have added comparisons to control numbers as follows:

Line 261: The viability rates of the fourth and fifth cross were 78% (n=100) and 79% (n=89) respectively, though this is still lower than the average viability of eggs from nine wildtype females (85% n=857) (S1 Table S1,S2 Table).

Line 236: “…somatic GFP expression…” Is amhy normally expressed in somatic tissue or is this expression due to influence of insertion site? amh is gonad specific in all other vertebrates.

Author Response: Thank you for this question. We have clarified that the fluorescence is from the constitutive Ef1a:EGFP cassette, not from the native promoter of *amhy*.

Line 244: One fertile male also produced offspring with somatic GFP expression at a very low frequency (7%, n=231), indicating a heritable germline integration of the transgene containing the constitutive Ef1a:EGFP cassette had occurred.

Line 337: “histology of these males…” It is not clear what males are being analyzed. Male offspring that have somatic GFP expression?

Author Response: We agree that this was unclear. We have specified which males we analyzed:

Line 247: Histology of F0 XX *Tg(amhy,EGFP)* males showed key differences between fertile, subfertile, and hypotrophic testes (Fig 3A)

Lines 230-243. This section seems out of place since rest of Fig. 3 is presented above (i.e. this section comes after Figure 4 presentation).

Author Response: Thank you for pointing this out. We have reorganized this section and moved the fertility data of XX transgenic males before data for XY knockout females. Now, all material from Figure 3 is presented before introducing material from Figure 4.

Figure 3: Please add wild-type XY age-matched testis for comparison with XX Tg(amhy;egfp) testes. Images are two small to assess stages of spermatogenesis (e.g. arrow to spermatogonia in Fertile panel is pointing to cells that appear to be outside the tubule/lobule, while spermatogonia would be expected to be within the tubule/lobule.

Author Response: We have added wildtype testis images to figure 3 and increased the magnification so that the gametes can be seen more clearly.

Line 251: This sentence is confusing because it starts off talking about mutants (“Neither amhy mutant…” but then appears to include transgenics [presumably the XX Tg(amhy,EGFP)]. Please make it clear what the genotypes of these fish are.Transgenics are usually not referred to as mutants unless the insertion site disrupts a gene.

Author Response: We have clarified the specific genotypes of interest as follows:

Line 305: Neither XX *Tg(amhy,EGFP)* males nor XY *amhy*-KO females appeared to fully recapitulate wildtype XY male coloration, but both were distinct from wildtype XX females (Fig 5A).

Line 278: Estimating gene expression levels/timing of amhy vs. amha (or any gonad-expressed gene) using bulk RNA seq on RNA isolated from whole fish to is problematic. At the time their expression initiates, the gonads and expected to be a very small proportion of the fish, and only a small number of cells/gonad may be expressing these genes. A more accurate approach would be to assay expression by RNA in situ hybridization on isolated gonads or paraffin sections were gonadal cells can be identified (or RT-qPCR on isolated gonads). This is especially important given that at these time points there appear to be no detectable differences in the expression of amha in males vs. females (more on this below).

Author Response: We agree that in the early primordial gonad only a small number of cells might be expressing *amhy,* which may be difficult to accurately measure when conducting RNA-seq on whole embryos. We were able to detect an increase in *amhy* expression at later developmental stages, which is consistent with a role in sex determination. However, we acknowledge this difference may be stronger if we were able to dissect early developing gonads only for RNA-seq (see earlier response highlighting the difficulty in this). We are currently assaying spatial expression patterns of *amh* and *amhy* relative to known somatic and germ cell markers using HCR *in situ* hybridization. We are aiming to use this approach to also quantify overall expression levels of *amhy* in the primordial gonad, but the timeline of these experiments are outside the scope of this manuscript. We have acknowledged the limitations of whole embryo data and highlighted these alternative approaches:

Line 522: While we do see substantial sequence divergence in the putative regulatory regions of amhy relative to amh, we only detected a significant difference in expression at a single developmental stage. As we are unable to isolate embryonic gonadal tissue for expression analysis, our results in this study are limited to characterizing total expression in the whole embryo. This likely reduces our ability to detect differences in the expression of genes like amh and amhy which are restricted to a small population of cells. Alternative approaches, like single cell RNA-seq, spatial transcriptomics, or HCR in situ will be useful to survey gene expression in the early differentiating gonad.

Line 282: It is stated that the sex determination likely begins before 3-4 days post hatch (or 11-12 dpf) since this is when differences in germ cell between males and females. However, referenced paper [81] shows detectable differences only starting at 15 dpf (reared at 19.5ºC), which would be ~7 dph. This later timing is consistent with reported increase in ddx4 and piwil1 starting at 8 dph, which is more consistent with sex determination occurring between 4-8 dph (not prior to 4 dph, as stated).

Author Response: Thank you for these observations and for taking the time to consider the Lewis et al. 2008 reference. While morphological differences are not observed until 15 dpf (~7 dph), there are differences in germ cell count observed at 11-12 dpf (3-4 dpf) noted in the section “Proliferation of PGCs beginning on page 915 and highlighted in Fig 9 on page 916. We have attempted to clarify this:

Line 352: The earliest identified difference between male and female threespine stickleback is an increase in primordial germ cell count in females relative to males at 3 to 4 dph, followed by differences in germ cell morphology at 7 dph [86].

For better comparisons between these studies, it will be necessary to know the details of the rearing conditions. Please add this to the M&M (water type, temperature, food, etc... Refer to Ref 81 for example).

Author Response: We have added this information to the methods section:

Line 672: All experiments were performed using fish crossed and reared in laboratory conditions. Fish were maintained in 3.5 ppt Instant Ocean Sea Salt (Spectrum Brands, Blacksburg, VA, USA) in reverse osmosis water at 18 °C and pH 8.0 and a summer photoperiod (16L:8D). After hatching, fry were fed freshly hatched Grade A Brine Shrimp (Brine Shrimp Direct, Ogden, UT, USA) twice daily.

Also, please consider using dpf instead of dph since this does not rely on the reader knowing when hatching occurs.

Author Response: We have clarified that hatching itself is a developmental stage in stickleback and other times after hatching are measured from this stage.

Line 350: … stage 26 larvae (0 days post hatching (dph), around 200 hpf), as well as larvae 4 and 8 dph.

Lines 305-346: It is a curious result that neither the classic male (dmrt1) or female (foxl2a) genes show sex biased expression at the time points assayed (≤8dph), a time point the authors presume is after initiation of sex determination. However, an alternative hypothesis is that under the rearing conditions used, sex determination has yet initiate. It would therefore be necessary to extent the timepoints to ≥8dpf to a point where dmrt1 and foxl2a expression becomes sexually dimorphic. It may be that at these later timepoints, amhy is more strongly expressed in XY animals, as was predicted.

Author Response: Though we do not see differential expression of these canonical sex differentiation genes, our corrections to expression levels of *amh* (outlined on page one of this document) show higher expression of *amhy* relative to *amh* in males at developmental stages where sex determination should be occurring. Consistent with this, we also found that total *amh* dosage (*amh + amhy)* was higher in males than in females across these stages. These results along with the differential expression of a few key gonadal genes previously mentioned (*hsd17b1, amhrII, piwil1, ddx4*) suggest that sex determination has initiated.

We agree that we need to investigate later timepoints to look for further evidence of transcriptional gonadal differentiation. These experiments are currently underway, but we think they warrant a much more comprehensive characterization across multiple later stages of development to fully investigate the timing of key sex differentiation genes alongside the details of morphological gonad development which are not well characterized in stickleback. Our findings will form the basis of a future manuscript detailing sex differentiation in threespine stickleback.

It would also be informative to do RT-qPCR on isolated adult gonads to determine if these gene are expressed sex-specifically at some point. If they are, then this further supports that the timepoints analyze are not late enough to capture when sex determination is actually happening.

We have highlighted that the canonical sex differentiation genes are differentially expressed in our study shown through *in situ* hybridization and included a reference showing differential expression of these genes in total transcriptomes of adult gonads.

Line 558: These genes show expected dimorphic expression patterns in adult gonads, shown both by our *in situ* data as well as gonadal transcriptomic data [122].

Line 342: “…hsd17b1, which plays a role in estrogen activation.” Should be “…estrogen synthesis.” as hsd17b1 converts E1 to E2.

Author Response: Corrected.

Reviewer #3: In this study, the authors found that the sex-determining gene in the threespine stickleback is the amhy gene, which arose by copy-and-paste duplication from an autosomal copy. This result in itself is not particularly novel. However, the part where the authors manipulated this gene to produce XX males and XY females and showed that the Y chromosome is important for spermatogenesis and development of nuptial coloration and that YY individuals are lethal is extremely important. This is because the function of the Y chromosome other than sex determination remains largely unknown in many taxa, and it is not known whether sexually antagonistic genes (e.g., genes that favor males and disfavor females) accumulate on the Y chromosome or to what extent harmful mutations accumulate on the Y chromosome. Therefore, I recommend acceptance of this paper after proper revision.

One major suggestion is that the authors should explain these topics (functions and deleterious mutations of Y chromosomes) in Introduction. That will make the novelty of this paper more clear.

Author Response: Thank you for this suggestion. We think this is a very exciting topic and have highlighted it in the introduction.

Line 82: Empirically testing which genes on sex chromosomes are sexually antagonistic remains challenging, as these genes are perfectly linked to the sex determination gene. Thus, evaluating the fitness effects of sex-limited loci in the opposite sex (i.e. Y-linked loci in females) is not possible in most systems. Novel systems where sex can be isolated from other loci on the sex chromosome would be helpful for testing these theoretical models of sex chromosome evolution.

Here are other suggestions and questions in the order of appearance;

L248 "develop throats": "develop red throats"?

Author Response: Corrected.

L254 "nuptial coloration": Which color do you mean, red throat, blue eye, or dark body?

Author Response: We have better described the elements of nuptial coloration in each genotype:

Line 308: XX males had blue coloration on their eyes and bodies, but none were as dark as completely colored XY males. XY females were varied in appearance, but some had discernably blue eyes and darkened bodies not seen in XX females.

L269 "less sequence identity": Can you show any number, such as % identity?

Author Response: Thank you for this suggestion. We have added sequence identity values into the text beginning on line 334. Subsequently, we have moved table 1 which previously contained this data to a supplementary table to conserve manuscript space.

L271 "consistent with weaker purifying selection acting on the Y chromosome due to a lack of recombination": I cannot follow this logic. What data is consistent with weaker purifying selection?

Author Response: We have clarified this section as follows:

Line 336: Lower sequence identity despite shorter divergence time is consistent with weaker purifying selection acting on the Y chromosome due to a lack of recombination

L272-273: According to Table 1, which dN/dS value is higher appears to vary among regions.

Author Response: Thank you for pointing this out. We have specified that the overall dN/dS is higher between threespine *amh* and *amhy* and highlighted that this pattern does vary across the exons.

Line 338: This was also supported by a higher overall dN/dS value between threespine *amh* and *amhy* (0.780) than between threespine *amh* and ninespine *amh* (0.668), though this pattern did vary across exons (S5 Table).

Fig. 5: These are images of just representative individuals. Can you get any quantitative data?

Author Response: We have quantified coloration and outlined our findings as follows:

Line 311: We measured the proportion of body area that was blue or dark for each genotype (S8 Fig, S4 Table). As red throats are not prominent even in wildtype males under our laboratory conditions, we were not able to quantify this trait. Both proportion of dark and blue body area showed wide variation within genotypes (Fig 5B). We saw significantly more blue (p < 0.0001) but not dark coloration (p = 0.1161) in XY males compared to XX females. XX males were significantly less blue (p = 0.0003) and dark (p = 0.0007) than XY males. XY females were significantly more blue than XX females (p = 0.0348), but there was no difference in darkness (p = 0.1074).
